# Ultrastructure of Ediacaran cloudinids suggests diverse taphonomic histories and affinities with non-biomineralized annelids

**DOI:** 10.1038/s41598-019-56317-x

**Published:** 2020-01-17

**Authors:** Ben Yang, Michael Steiner, James D. Schiffbauer, Tara Selly, Xuwen Wu, Cong Zhang, Pengju Liu

**Affiliations:** 1 Institute of Geology, Chinese Academy of Geological Sciences, Beijing, 100037 China; 2 Department of Earth Sciences, Freie Universität Berlin, Berlin, 12249 Germany; 3 Department of Geological Sciences, University of Missouri, Columbia, Missouri 65211 USA; 4 X-ray Microanalysis Core Facility, University of Missouri, Columbia, Missouri 65211 USA; 5 Laboratory of Marine Organism Taxonomy and Phylogeny, Institute of Oceanology, Chinese Academy of Sciences, Qingdao, 266071 China; 6 School of Earth Science and Engineering, Shandong University of Science and Technology, Qingdao, 266590 China

**Keywords:** Precambrian geology, Stratigraphy, Palaeontology

## Abstract

Cloudinids have long been considered the earliest biomineralizing metazoans, but their affinities have remained contentious and undetermined. Based on well-preserved ultrastructures of two taxa, we here propose new interpretations regarding both their extent of original biomineralization and their phylogenetic affinity. One of these taxa is a new cloudinid from Mongolia, *Zuunia chimidtsereni* gen. et sp. nov., which exhibits key characteristics of submicrometric kerogenous lamellae, plastic tube-wall deformation, and tube-wall delamination. Multiple carbonaceous lamellae are also discovered in *Cloudina* from Namibia and Paraguay, which we interpret to have originated from chitinous or collagenous fabrics. We deduce that these cloudinids were predominantly originally organic (chitinous or collagenous), and postmortem decay and taphonomic mineralization resulted in the formation of aragonite and/or calcite. Further, based on our ultrastructural characterization and other morphological similarities, we suggest that the cloudinids should most parsimoniously be assigned to annelids with originally organic tubes.

## Introduction

The onset of metazoan biomineralization is a key innovation in the evolutionary history of animals. *Cloudina* and other contemporaneous cloudinids have been widely accepted as the earliest biomineralized metazoans^1,2^, although varied preservation and influences of diagenetic alteration are evident^1,3,4^. *Cloudina* is most often considered a calcareous biomineralizer, with interpretations of its primary composition ranging from high-Mg calcite-impregnated organic material^1^ to aragonite^4^. More recently, the tubes of *Cloudina* have been interpreted as a product of crystallization by particle attachment of amorphous calcium carbonate^5^. Interpretations also vary on the degree of extracorporeal tube rigidity, either building those that were robust and unbendable^2^ or instead delicate and flexible^1^.

Interpretations on the affinity of cloudinids, and especially on *Cloudina*, have been controversial since its first descriptions. Most studies have focused on comparisons of gross morphology with similar tubular constructions in either annelids (serpulids in particular^3^) or tube-forming cnidarians^3,6,7^. Partly based on both the premise of its biomineralization and inferred affinity with anthozoan-like cnidarians^8,9^, recent studies have implicated *Cloudina* as an early reef-builder, although this interpretation has been challenged^10^. The abrupt disappearance of cloudinids at the Ediacaran-Cambrian (E-C) boundary has been traditionally considered a mass extinction and major faunal turnover^11^. In contrast, recent studies have demonstrated that cloudinids may have persisted into the early Cambrian^12,13^.

Despite uncertainties surrounding biomineralization and biological affinity, there is a broad consensus that Ediacaran tubular fossils, and specifically those of the cloudinids, are among the earliest skeletal metazoans^1,3,14–17^. Detailed investigations of their skeletal architecture and comparative affinity are therefore imperative for reconstructing early metazoan biomineralization and evolution. Supplemented with a review of taphomodes in cloudinids, we provide here the first report of cloudinids from Mongolia as well as a detailed investigation on the ultrastructures of well-preserved *Cloudina*. This work yields new information on the state of biomineralization and biological affinity of the cloudinids.

## Results

### Field observations

**Figure 1:**
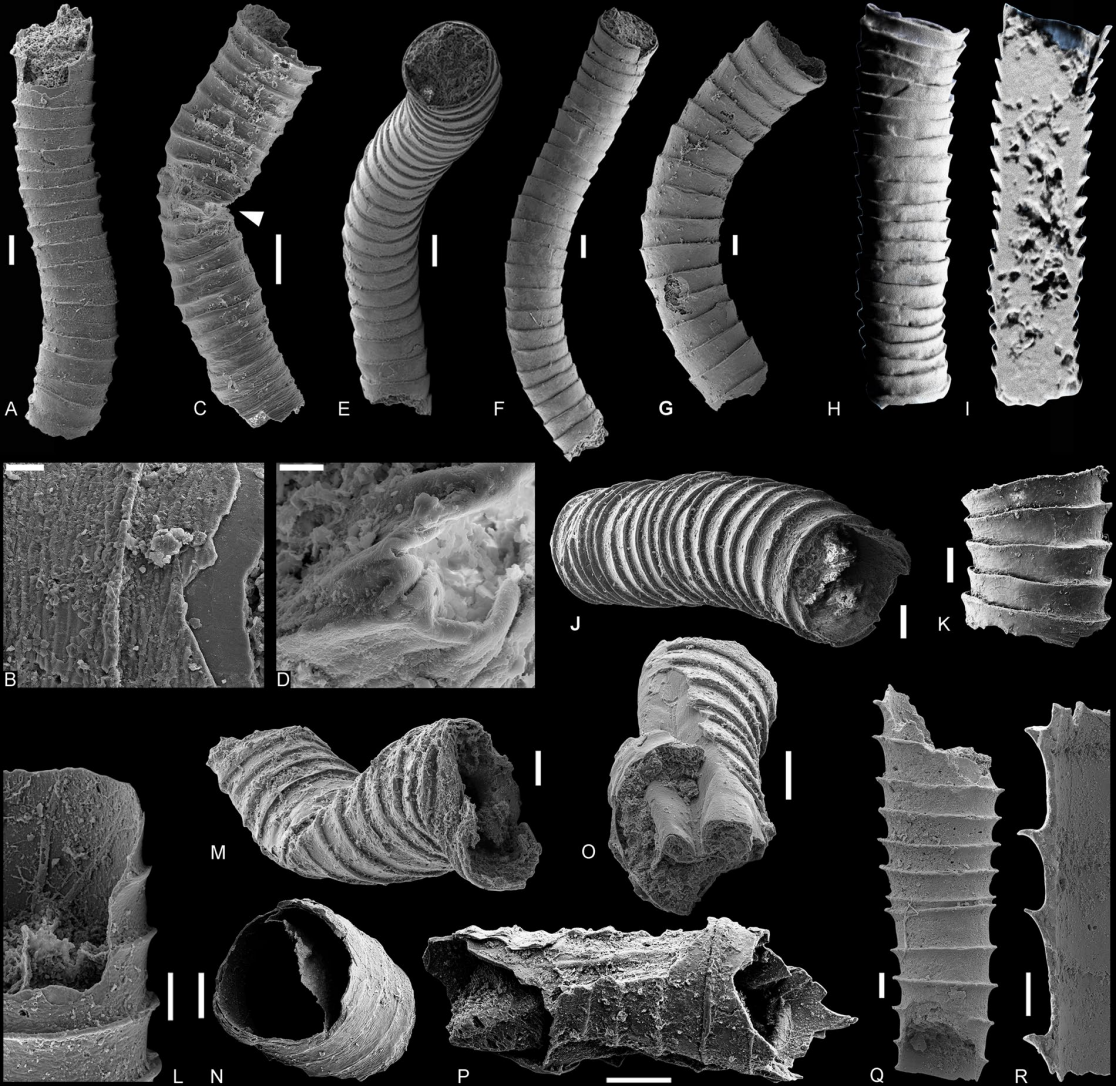
New cloudinid assemblages from Bayan Gol (**A–P**) and Salany Gol (**Q,R**) of Zavkhan Block, western Mongolia (see Supplementary Fig. 1 for the information of locality and stratigraphy), including *Zuunia chimidtsereni* gen. et sp. nov. (**A–P**) from the basal Zuun-Arts Formation, and *Rajatubulus* sp. (**Q,R**) from the upper Salanygol Formation. (**A**) Holotype, BGolN68Gl-07. (**B**) Close-up of (**A**) shows collar characteristics. (**C**) Paratype, BYN1108. (**D**) Close-up of C (white arrow) shows plastic deformation. (**E**) Paratype, BGolN68Gl-01. (**F**) Lateral view of (**E**). (**G**) Paratype, BGolN68Gl-19. (**H,I**) NanoCT-based volume model of *Z*. *chimidtsereni* gen. et sp. nov., BGolN68-4-6. (**I**) Half volume nanoCT-model of the specimen as in (**H**). (**J**) SEM image of (**G**) showing typical collared structures of cloudinids. (**K**) Lateral view of *Zuunia* n. gen. showing a typical collared structure of cloudinids, BGolN68-011. (**L**) Specimen showing fine rugae and smooth lumen, BGolN68Gl-12. (**M**) Distal view of the specimen showing plastic deformation, BGolN68aW-05. (**N**) Specimen showing delamination, BGolN68-013. (**O**) Paratype, indicating deformation and delamination of the tube, BGolN68Gl-10. **(P**) Specimen showing delamination, BGolN68-015. (**Q**) *Rajatubulus* sp., SAL127-003. (**R**) Longitudinally fractured *Rajatubulus* sp. showing a typical collared structure of cloudinids, SAL127-006. Scale bars: (**D**), 10 μm; (**B**), 20 μm; (**L**), 50 μm; (**E,F**), 200 μm; others, 100 μm.

Sediment sequences from the critical E-C transition are widespread on various blocks of Western Mongolia. The fossil assemblages described here were recovered from the basal Zuun-Arts Formation (late Ediacaran) in Bayan Gol and the upper Salanygol Formation (Cambrian Stage 3) in Salany Gol, Zavkhan Block, Mongolia (Supplementary Fig. 1). The basal Zuun-Arts Formation contains 10–20 m of shales interlayered with thin limestone, overlying the columnar stromatolites of the Shuurgat Formation with an erosive boundary^18^. The new fossils reported here (Fig. 1A–P) are preserved within the limestone interlayers of the shales, ~5 m above the lower boundary of the unit. Overlying the fossiliferous units but still within the Zuun-Arts Formation are microsparitic ribbonites and rhythmites^18^. Although previous work has reported the occurrence of small shelly fossils, including *Anabarites trisulcatus*, from the Zuun-Arts Formation^19^, subsequent investigation, including this one, failed to recover these Cambrian fossils^18^.

The position of the E-C boundary in Mongolia has been disputed, largely because the index fossil *Treptichnus pedum* has only been documented from the younger interval of the middle Bayangol Formation^18,20,21^. Previously, a significant negative δ^13^C excursion (anomaly “W”)^21^ in the middle-upper Zuun-Arts Formation was utilized to assess the position of E-C boundary. Smith *et al*. confirmed the existence of the “W” anomaly in the middle Zuun-Arts Formation; however, they also recognized a larger negative carbon isotopic excursion at the top of the Zuun-Arts Formation, closer to the first appearance datum of *Anabarites trisulcatus*. Due to the absence of high-resolution biostratigrapic data, we herein adopt the Smith *et al*. model, designating the first strong negative carbon isotopic excursion as the basis for the position of the E-C boundary in Mongolia (Supplementary Fig. 1B). However, it has been noted elsewhere^22^ that the internationally applied concepts for the definition of the E-C boundary require a formal re-evaluation. The newly reported Ediacaran tubular fossils occur in the basal Zuun-Arts Formation underlying the beds with anomaly “W”. Overlying the Zuun-Arts Formation, the Bayangol Formation consists of diverse carbonate beds interlayered with siltstones and hosts the first assemblage of Cambrian small shelly fossils^23^. Taken together, these fossils along with the reported carbon isotope excursions may help to provide a more complete chemo- and biostratigraphic framework for the E-C transition in Mongolia.

**Figure 2:**
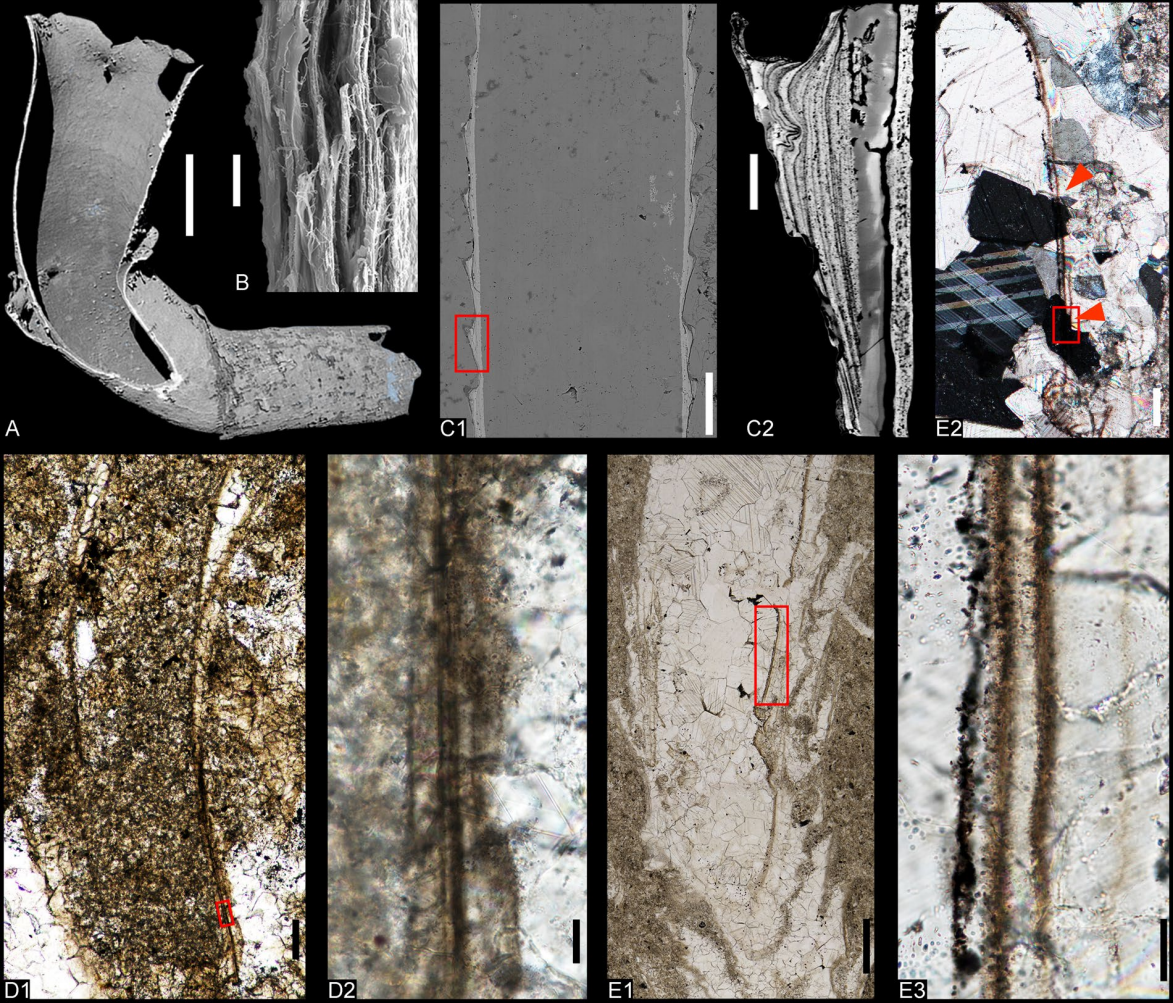
Lamellate structures of cloudinids and modern siboglinids. (**A**), nanoCT-based volume model of modern siboglinid, *Tevnia* sp. with collared tube, MTES-01. (**B**), SEM micrograph showing chitinous lamellae of the tube wall of Modern *Arcovestia ivanovi*, Arc3-04. (**C1**) SEM micrograph of cross section of *Zuunia* gen. nov., BYN1101. **(C2**) Backscattered electron (BSE) image of one collar (**C1**) showing a fine lamellate, submicrometric construction, uncoated. (**D1**), light micrograph of *Cloudina hartmannae* from Farm Aar, Namibia, A801K. (**D2**), close-up of **D1** (red frame) showing multiple fine lamellae and large blocky spar crystallites partly extending from the wall into the surrounding carbonate. (**E1**), light micrograph of *Cloudina hartmannae* from Paraguay, PGC010606. (**E2**), close-up of (**E1)** (red frame) with crossed nicols showing calcite spars (red arrows) extended across the layers of the wall. (**E3**) close-up of (**E2)** (red box) showing fine organic lamellae. Scale bars: (**A**), 2 mm; (**B**), 10 μm; (**C2,D2,E3**), 20 μm; **(C1,D1,E2**), 100 μm, **E1**, 500 μm.

**Figure 3:**
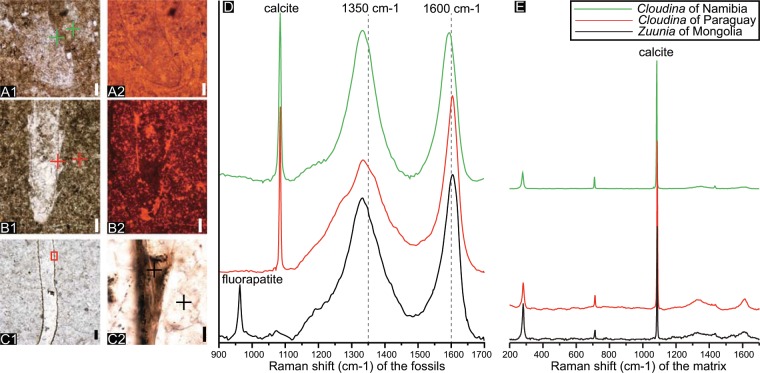
Analysis of Raman spectroscopy (**D,E**) and cathodoluminescence (CL; **A2**–**C2**) on *Zuunia* gen. nov. and *Cloudina*. (**A**), *Cloudina hartmannae* from Farm Aar of Namibia showing organic wall in transmitted light (**A1**) and CL (**A2**), A0120. CL image reveal an undifferentiated calcification of the whole specimen. (**B**), *Cloudina hartmannae* from Paraguay showing organic wall in transmitted light (**B1**) and CL (**B2**), pgc010201. CL analysis reveals four carbonate generations randomly distributed in the fossil and matrix. (**C1**), Longitudinal section of *Zuunia* gen. nov., BYN11b0101. (**C2**), A collar of (**C1**, red frame) showing lamellae. (**D**), Raman spectra showing prominent amorphous carbon bands in the wall of the fossils (at 1350 cm^−1^ and 1600 cm^−1^). (**E**) Raman spectra showing calcitic bands of the matrix (right graph). Crosses in the micrographs (**A–C**) mark the spots of Raman spectroscopic analyses. Scale bars: (**C2**), 10 μm, others 200 μm.

In addition, another cloudinid, *Rajatubulus* sp. (Fig. 1Q,R), was also recovered from the topmost carbonates of the Cambrian Stage 3 Salanygol Formation of the Salany Gol section. For comparison, *Cloudina* specimens (Figs. 2D,E, 3A,B) from the Mooifontein Member of Nama Group (Farm Aar, South Namibia), and Tagatiya Guazu Formation (Northeast Paraguay), were also assessed.

### New cloudinid assemblages

The Ediacaran Zuun-Arts assemblage is a low-diversity assemblage, containing collared tubes of *Zuunia chimidtsereni* n. gen. et sp. (Fig. 1A–P) fossils and egg stages.

The assemblage of the uppermost Salanygol Formation yields *Rajatubulus* sp. (Fig. 1Q,R) co-occurring with a diverse suite of Cambrian Stage 3 small shelly fossils, such as *Lapworthella, Camenella*, and *Latouchella*. These younger cloudinids have previously only been found in the early Cambrian of Kazakhstan^12^.

### Systematic palaeontology

Annelida Lamarck, 1809^24^

**Genus** *Zuunia* gen. nov.

Type species: *Zuunia chimidtsereni* gen. et sp. nov.

Etymology. The name is derived from the Zuun Arts Mountain, 65 km southwest of Uliastai, Mongolia.

#### Diagnosis

Same as for the species

*Zuunia chimidtsereni* gen. et sp. nov.

#### Etymology

The species name is in honor of the renowned geologist Anaad Chimidtseren (Mongolian University of Science and Technology), who supported our study in many aspects.

#### Type-material

Holotype, BGolN68Gl-07 (Fig. 1A). Paratypes, BYN1108 (Fig. 1C), BGolN68Gl-01 (Fig. 1E), BGolN68Gl-19 (Fig. 1G), BGolN68Gl-10 (Fig. 1O). BGolN68Gl-01, -07, -08, -19, and -10 are hosted in the Department of Geoscience, Freie Universität Berlin (FUB). BYN1108 is hosted in the Institute of Geology, Chinese Academy of Geological Sciences (IGCAGS).

#### Diagnosis

**Figure 4:**
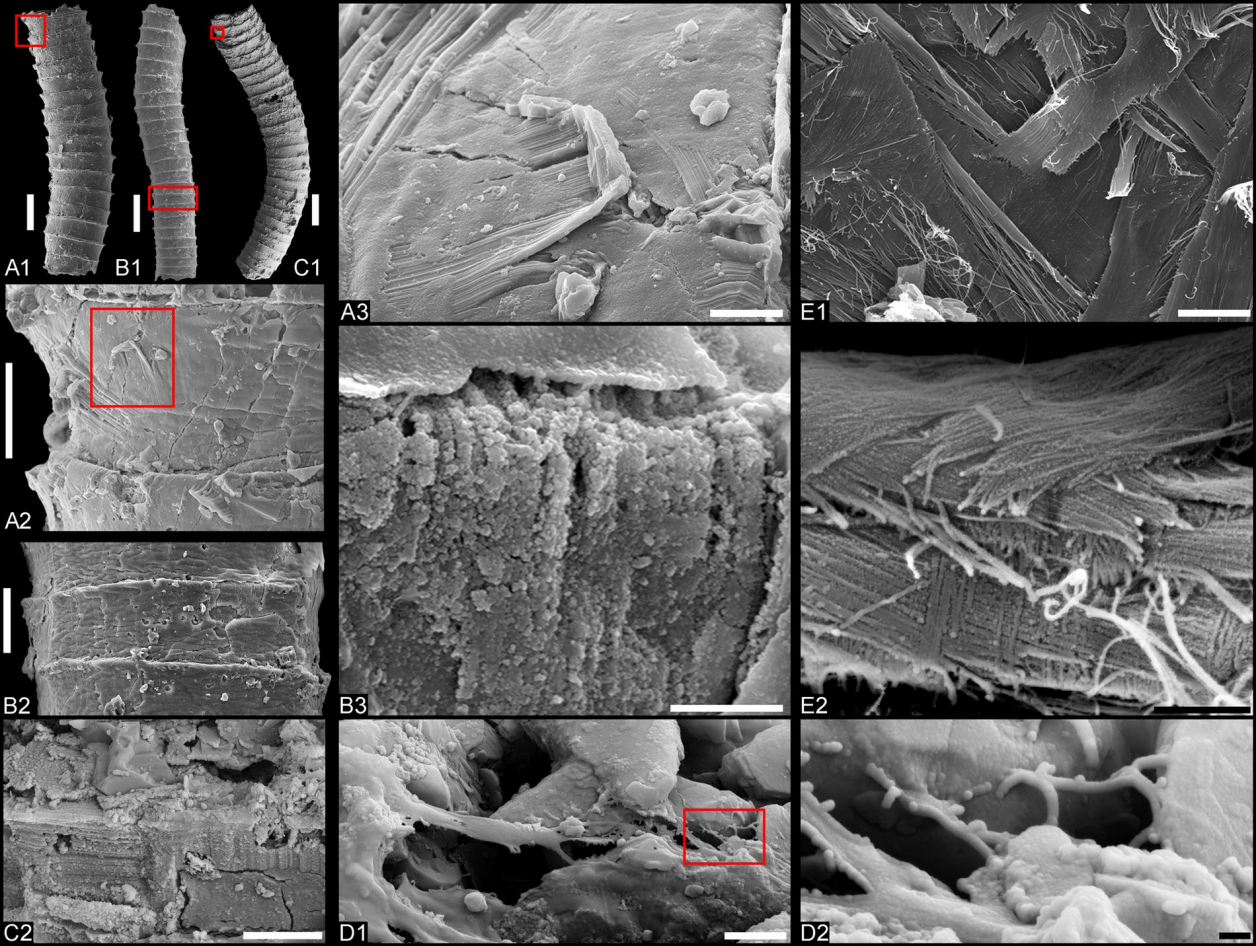
Natural pseudo-orthogonal plywood fabrics and fibrous ultrastructures of tube wall in *Zuunia* gen. nov. (**A–D**) and modern siboglinid *Arcovestia ivanovi* (**E**). Images of *A*. *ivanovi* were taken from the specimen figured in Fig. 6B. (**A**), *Zuunia* gen. nov. showing pseudo-orthogonal natural plywood structures of phosphatized fibers (**A3**), BYN1203. (**A1**), overview of tube. (**A2**), close-up of A1 (red frame). (**A3**), close-up of A2 (red frame). (**B**) *Zuunia* gen. nov. with fibrous structures at naturally eroded sites of the wall, BYN1202. (**B1**), overview of tube. (**B2**), tube with rugate surface ornamentation, close-up of B1 (red frame). (**B3**), close-up of B2 (red frame). (**C**), *Zuunia* gen. nov. (**C1**) showing pseudo-orthogonal natural plywood structures of phosphatized fibers (**C2**), BGol68aW-07zd. (**C1**), overview of the tube. (**C2**) close-up of (**C1**) (red-frame). (**D1**) organic lamella, partially retaining nano-fibril texture, released from tube etching with hydrochloric acid, BGolN68Etch-04. (**D2**) close-up of organic fibrils, seen in **D1** (red frame). (**E1**) micrograph of chitin fibers on outer tube surface of *Arcovestia ivanovi* arranged in a pseudo-orthogonal natural plywood pattern. (**E2**) close-up of the chitin fibre arrangement in **E1**. Scale bars: (**A1–C1**), 200 μm; (**A2**), 25 μm; (**B2**), 50 μm; (**A3,E1**), 50 μm; (**B3,C2**), 5 μm; (**D1,E2**), 2 µm; (**D2**), 200 nm.

Slender tubular fossils with low divergent angle and regularly developed collars along the longitudinal axis, which do not flare widely. The outer surface is ornamented with fine rugae (Fig. 1B), while the inner surface of the wall is smooth (Figs. 1L, 2C1, 3C1). The cross section is circular. The tube wall is composed of multiple thin organic lamellae, composed of nanometric fibrillae (Fig. 4).

#### Description

**Figure 5:**
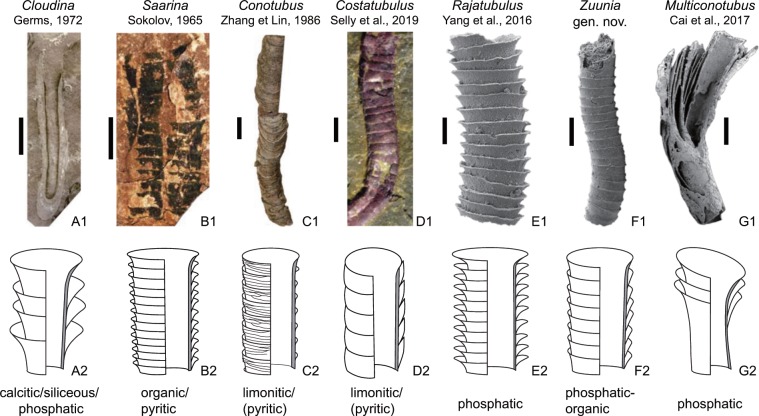
Comparison of morphologies and taphonomic modes within the cloudinid morphoclade. The top row is representative specimens of fossils (A1–G1) with sketches underneath showing schematic differences (A2–G2). (A1) Holotype of *Cloudina hartmannae* Germs, 1972 (Photo courtesy of the Iziko Museum of South Africa). (B1) Holotype of *Saarina juliae* Gnilovskaya, 1996 (Photo courtesy of Natalia Bykova). (C1), Syntype of *Conotubus hemiannulatus* Zhang et Lin, 1986. (D1) Part of the holotype of Costatubus bibendi, Selly *et al*., 2019. (E1) *Rajatubulus costatus* from Kazakhstan (modified from^12^, Fig. 7B). (F1) Holotype of *Zuunia chimidtsereni* gen. et sp. nov. (Fig. 1A). (G1) *Multiconotubus chinensis* Cai *et al*., 2017 (modified from^25^, Fig. 8B). Scale bar: (E1) 100 μm; (F1), 200 μm; (A1,D1,G1), 1 mm; (B1,C1), 5 mm.

The Mongolian cloudinids are small, mostly fragmented tubes with a length of up to 5 mm, diameter of 0.1–0.8 mm, and tube wall thickness of ~15–25 µm. The collared structure of the tubes indicates a similar mode of construction as in the other cloudinids. In contrast to *Cloudina*, however, the collars are highly regular and do not flare as widely as in *Cloudina, Saarina*, or *Rajatubulus* (Fig. 5; Supplementary Figs. 1, 2). The tube surface shows a characteristic rugate ornamentation (Fig. 1B), similar to *Cloudina hartmannae*^25^, while the inner surface of the wall is non-textured, indicating a smooth lumen (Figs. 1L, 2C1, 3C1). The tubes are taphonomically phosphatized, but often show plastic deformation (Fig. 1C,D,M). Ultrastructurally, the tube walls are finely lamellate in construction (Figs. 2C2, 3C2). Inner lamellae of the tubes are occasionally delaminated and crumpled inward (Fig. 1N–P). Both energy dispersive x-ray spectroscopy (EDS) and Raman spectroscopy show the presence of carbonaceous matter within the wall (Fig. 3D, Supplementary Fig. 3).

#### Occurrence

Basal Zuun-Arts Formation of Bayan Gol, western Mongolia, late Ediacaran.

### Ultrastructures and Raman spectroscopy

Petrographic analysis of thin sections and scanning electron microscopy (SEM) revealed the presence of a lamellate ultrastructure structure of the tube wall of *Zuunia* n. gen. (Fig. 2). Up to 10 individual organic lamellae have been recognized in backscattered electron imaging and with energy dispersive x-ray spectroscopy (EDS; Fig. 2B, see also Supplementary Fig. 3). The organic lamellae are usually thin (mostly <1 µm, with rare exceptions up to 2 µm), with intercalated layers of granular apatite.

Naturally weathered surfaces of *Zuunia* n. gen. tubes reveal the presence of phosphatized fibrils overlying each other in pseudo-orthogonal, plywood-like fabrics (Fig. 4A–C). Similar textures can be seen in the arrangement of collagenous fibers of cortical bandages of graptolites^26^ and the chitinous fibers of modern polychaete tubes (Fig. 5E)^27^. Organic films were released from the phosphatic tubes of *Zuunia* n. gen. by etching in dilute hydrochloric acid (Fig. 4D). The films have a rubbery ultragranular consistency interpreted to result from kerogenization of the original organic matter. At their margins, the released organic films sometimes preserve a fibrous structure with single 80–200 nm-diameter fibers (Fig. 4D2).

We additionally document comparable organic lamellae (up to eight) in single wall layers of *Cloudina* specimens from Namibia (Figs. 2D, 3A) and Paraguay (Figs. 2E, 3B). In thin-section, the carbonaceous lamellae have a granular appearance, probably due to a combination of maturation of the original organic matter and recrystallization of the calcite during diagenesis. The thickness of the organic layers (0.5–5 µm in Namibia; 0.5–8 µm in Paraguay) is greater than those observed in *Zuunia* n. gen. In both *Cloudina* and *Zuunia* n. gen., these organic lamellae are discernible in each wall of the episodically grown collared segments. The organic lamellae in *Cloudina* are partly embedded in large calcite spars that sometimes extend into the lumen infill or external carbonate sediments (Fig. 2E2). Our observation more clearly points to a diagenetic origin of the calcite spars in the tube wall^1,4^, and does not support notions that the walls consist of micritic primary layers fusing to secondary laminae^7,28^.

Raman spectroscopic analysis of the walls of *Zuunia* n. gen. (Fig. 3C) and *Cloudina* (Fig. 3A,B) provide clear evidence for amorphous carbonaceous material with two broad bands at ~1350 cm^−1^ (D1-, D4-band) and ~1600 cm^−1^ (D2-, G-band), alongside subordinate bands of calcite and/ or fluorapatite. Measurements within the matrix reveal only the typical bands of calcite (Fig. 3E). The peak topologies of the carbonaceous matter in *Zuunia* n. gen. and *Cloudina* mostly indicate low-to-moderate maturity^29^.

Although cathodoluminescence examination of *Cloudina* thin sections from Paraguay indicate at least four different generations of calcite, these luminescent generations are randomly distributed throughout. No single generation strictly is confined to the tube wall, indicating multiple diagenetic recrystallization events (Fig. 3B2). *Cloudina* from Namibia shows a homogeneous luminescence of calcite spar generation within the tube wall, although this generation is also represented in patches of the surrounding carbonate sediments (Fig. 3A2).

### Cloudinid morphoclade

**Figure 6:**
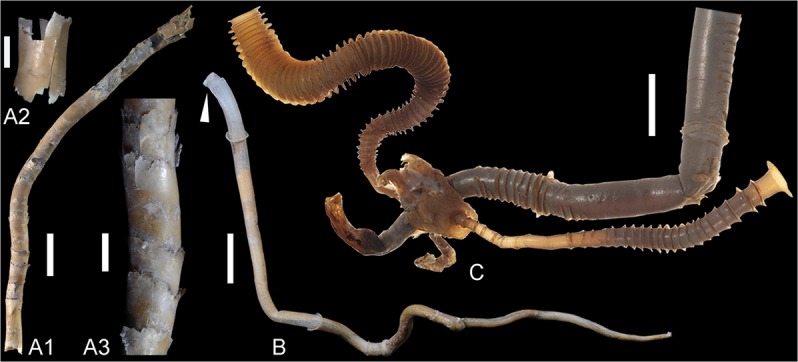
Modern siboglinid tubes showing varied collar-morphology and -spacing. (**A1**) *Alaysia* sp. from the Okinawa Trough, No. 194A5321. (**A2**) fragment of the tube, broken off from (**A1**), to experimentally show brittle fracture behaviour. (**A3**) close-up view of (**A1**) showing collars close to the wall. (**B**) *Arcovestia ivanovi* from the Manus Basin showing sparse collars, arrow marks position where fibrous ultrastructure is documented in Fig. 4E; Specimen No. Arc3-04. (**C**) *Tevnia jerichonana* indicating considerable variation of collar morphology and spacing, 400407-DSP (Image courtesy of Smithsonian Institution, National Museum of Natural History, Office of Education and Outreach, Cat. No. EO 400407). Scale bars: (**A2, A3**), 3 mm; others 10 mm.

The cloudinids are here viewed as a morphoclade united by episodic secretion of extracorporeal tubes with repetitive collars and a smooth internal tube lumen. These tubes are interpreted to have served two primary purposes: (1.) functioning as support structures that allowed for elevation of the organism above the sediment-water interface for access to nutrients; and (2.) providing protection from predation and water turbulence. This group of form-taxa (Fig. 5) includes *Cloudina*, *Saarina*, *Conotubus*, *Costatubus*^30^, *Multiconotubus*, and *Rajatubulus*, as well as potentially the undetermined *Cloudina*-like tubes in the Jurassic Figueroa deposit^31^^: fig. 15D^. Here, we place *Zuunia chimidtsereni* n. gen. et sp. within the cloudinid morphoclade, and consider it to be plausibly phylogenetically related to other cloudinids because it shares a similar morphology, construction, and multilamellar composition. The variable taphomodes, common diagenetic alteration of the strata, and the lack of ultrastructural details for many species of this morphoclade make it problematic to assign close phylogenetic relationships between these taxa. The ratio of collar width and spacing are of potential interest for the differentiation of taxa and show a linear correlation in *Zuunia*, *Rajatubulus*, and *Cloudina* (Supplementary Fig. 4), though it needs to be emphasized here that morphological characters such as size, spacing, angle, and width of the collars in modern tubular housings (e.g. siboglinids) are highly variable, even within single individuals (Fig. 6C; Supplementary Fig. 5). Therefore, although such characters have been used for palaeontological species description in the past, taxonomy solely based on morphological characters needs to be critically reviewed and re-evaluated for most members of the cloudinid morphoclade. On the other hand, ultrastructural details may provide a fundamental novel way forward for resolving such phylogenetic complications.

## Discussion

### Taphonomy and biomineralization

Cloudinids show a wide range of taphomodes, such as phosphatic, organic/pyritic, calcareous, limonitic/pyritic, and siliceous preservation (Fig. 5). More specifically, *Cloudina* itself has been documented with calcitic^1,2,28^, phosphatic^3^, and siliceous tube compositions^14,32^. Of these varying taphomodes, phosphatization and silicification are broadly considered to be secondary replacement of pre-existing carbonate^1,2,7,28^, while the possibility of phosphatization of an organic exoskeleton has been unexplored. Indeed, it has been widely accepted that phosphatization in the cloudinids, as well as in most Cambrian small shelly fossils, is of early diagenetic origin. However, phosphatization of other E-C fossils, such as exceptionally preserved embryos^33,34^ as an example, often replaces originally organic structural compounds rather than carbonate. Similar to *Rajatubulus* and *Multiconotubus*, *Zuunia* nov. gen. are preserved as phosphatized lamellate material. The delamination structures in *Zuunia* nov. gen. (Fig. 1N–P) support the interpretation of an organic tube construction with early diagenetic phosphatization.

While disputes have arisen over the composition of the biomineral component in cloudinid tubes—variously interpreted as aragonite, calcite, or high-Mg calcite^1,4,17^—the extent of their original mineralization is also in question. Previous studies have concluded that the calcitic shells of *Cloudina* are composed of neomorphic spar^1,4^, and this is also supported by our observations. The main reason for postulating the existence of an originally mineralized shell was the observation of “brittle deformation” (brittle fracture) of the tube walls^1^. However, brittle fracture is also observed in organisms with entirely unmineralized exoskeletons, such as the chitinous tubes of modern siboglinids, which show comparable features when subjected to compaction (Figs. 6A2 and 7). Indeed, it is well known that chitinous shells can vary from being non-brittle to highly brittle depending on the type, texture, and composition of the chitin-protein fibers^35^. The tube walls of *Cloudina* have been interpreted in some studies as containing an organic component within a biomineral structure^1–3,15,17^, largely owing to observations of plastic, as opposed to brittle, tube deformation^1,36,37^. Nonetheless, organic material has not been firmly documented prior to this study, although organic preservation and/or plastic deformation have been reported in other cloudinids^38–40^.

**Figure 7:**
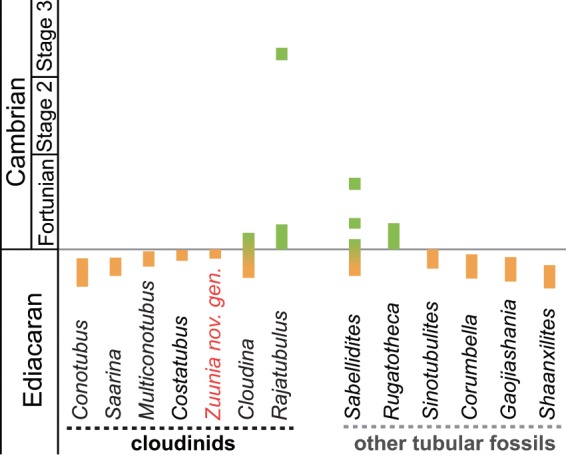
Temporal distribution of Ediacaran-Cambrian tubular fossils.

It is often complicated to determine whether existing skeletal mineralization in palaeontological samples formed *in vivo* or during diagenesis. Calcium isotopic studies have convincingly argued that the calcitic shell material of *Cloudina* and later fibrous cement generations have been neomorphosed from precursor aragonite^4^. However, the study failed to demonstrate whether the aragonite was a primary biomineral or an early diagenetic product of a decomposing organic shell. It has been documented that some modern chitinous siboglinid worm tubes undergo post-mortem aragonite replacement from microbial decay of tube wall proteins^41^. Whilst part of the ultrastructure is obliterated by this replacement, many subtle lamellae of former chitinous constructions are successfully preserved^41^. If modern siboglinids are an apt analogue, it is possible to conclude that aragonitic lamellae may have formed in *Cloudina* entirely during post-mortem diagenesis.

Our investigation of *Zuunia chimidtsereni* n. gen. et sp. reveals that the tube wall is finely lamellate, comprised of dense calcium phosphate interlayered with carbonaceous materials (Figs. 2C2, 3C, Supplementary Fig. 3). The organic-rich layers are kerogenized, but partially retain a microfibrillar construction (Fig. 4D). Fibrillar pseudo-orthogonal structures are also distributed in multiple layers of the phosphatized tube wall (Fig. 4A–C). The fibrils match the size ranges of typical structural biopolymers, such as chitin, cellulose, and collagen, which are widely utilized in the construction of metazoan exoskeletons (Fig. 4E). Raman spectra and EDS data of the lamellae in both *Zuunia* and *Cloudina* suggest the existence of multiple original organic layers (Fig. 3D, Supplementary Figs. 2, 3). The phosphatic lamellae are considered to be early diagenetic impregnation. We conclude that the tubes of *Zuunia* nov. gen. were primarily composed of chitin or collagen.

Although the ultrastructure of other cloudinid genera has not been well investigated, or instead not retained during replacive mineralization processes^42^, the organic components in the wall of *Cloudina*^16^ have been inferred, and are herein illustrated for the first time. Phosphatized tubes of *Cloudina* from South China have revealed a lamellar construction and partial delamination of inner tube lamellae^16^, revealing that they were likely not primarily phosphatic. The granular ultrastructure reported from phosphatic remains of *Cloudina*^16,43^ likely reflects diagenetic apatite crystallites which cannot be directly compared to the aragonite/calcite ultrastructure of modern serpulids, as was stressed by a previous study^6^. Investigation on the original composition of the Ediacaran tubular fossils remain in the early stages, although morphological observation can provide complementary support towards the interpretation of their primary composition. In short, the tubes of *Cloudina* display inconclusive evidence for an original, primary carbonate composition. Instead, the organic lamellae and plastic deformation logically indicate an organic primary composition. Carbonaceous taphomodes in *Conotubus*^39^, *Saarina*^40^, *Shaanxilithes*, the chitin-like fibers in the tube walls of *Sabellidites*^38^, the mixed plastic and brittle fracture in calcareous tubes of *Corumbella*^44,45^, and the lamellate *Sinotubulites*^16^, may in sum suggest that many terminal Ediacaran tube-forming metazoans, if not all, developed lamellate organic housing constructions.

### Affinity

Even with decades of investigation, the biological assignment of cloudinids remains highly contested. Contributing to this uncertainty is their strong diagenetic alteration and diverse preservational modes. Most phylogenetic efforts have focused on *Cloudina*, though recent studies have recognized that the cloudinid morphoclade embraces a number of taxa with similar construction but various preservational modes^12,25^. *Cloudina* has been interpreted as either an alga^46^ or as an eumetazoan^14^ —the latter with specific affinities, including cribricyathean archaeocyaths^17^, anthozoan-like cnidarians^6^, and several annelid designations^3,16,47^. Both Hahn and Pflug^47^ and Conway Morris *et al*. chose not to shoehorn *Cloudina* into any known taxonomic level, instead supporting a designation as an *incertae sedis* metazoan family, the Cloudinidae.

Comparing *Zuunia* gen. nov. with other taxa reveals that the tube constructions of the morphoclade are defined by variable collars, and a smooth inner lumen. Such collared constructions can be observed in hemichordate pterobranchs and various families of tube-dwelling annelids, but not in tube-forming cnidarians. While lamellar microstructures widely exist in metazoan exoskeletons, the plywood-like texture of structural fibrillar compounds is comparatively restricted. It has been described from the cuticles of beetles^48^, as well as from housing constructions of graptolites (pterobranchs) and annelids^26,27,49,50^. The coincidence of tube morphologies and pseudo-orthogonal organization of fibrillar compounds in graptolites and polychaetes is remarkable, however, likely due to a comparable mode of tube secretion and construction^27,51^. There are, of course, morphological differences in their construction, as pterobranch tubes are constructed by fusellar half-rings, but such features are absent in cloudinid tubes. Instead, the ultrastructure and tube construction in *Zuunia* n. gen. is much more comparable to tubicolous annelids, such as the tubes of siboglinids and alvinellids, which are entirely organic (chitinous and proteinaceous) in composition (Figs. 2A,B and 4E)^52^. It is worth noting that many annelid tubes contain a high phosphylated protein content, important for adhesiveness in the tube construction^53^. These proteins are capable of binding calcium, and thus may have played a pivotal role for the early diagenetic phosphatization pathway, which has been repeatedly documented in many Ediacaran tubular fossils such as in cloudinids and sinotubulitids.

Tubular exoskeletons with certain similarities to the cloudinids are also common in modern scyphozoan, hydrozoan, and anthozoan cnidarians. While fibrillar chitin is widely distributed in the exoskeletons of scyphozoans and hydrozoans^54^, it plays a subordinate role in the formation of organic tubes, where irregular networks of collagenous ptychocysts form a coarser organic network^55^. A lamellar construction is also seen in scyphozoan tubes (Supplementary Fig. 2A)^56–58^ as well as in hydrozoan hydrothecae (Supplementary Fig. 2C). Chitinous fibers are often indistinguishable in the hydrotheca and are commonly glued by other organic compounds, but some irregularly ordered lamellae of chitin fibers can be recognized in the hydrocaulus (Supplementary Fig. 2B4). This type of lamellar and fibrillar construction is less ordered than in modern siboglinids, and can be distinguished from the investigated cloudinids. Although some annelids have developed various modes of tube secretion, including agglutination, secretion of loose fibrillar lamellae^27^, or the unique chevron-shaped growth structures of the biomineralized serpulid tubes^6^, all of which are distinct from the lamellar tubes of *Zuunia* n. gen. We conclude that the morphology and its fibrous ultrastructure justifies a consideration of an annelid affinity for *Zuunia* n. gen.

It has been argued that reports of basal terminations^3,59^ and branching/budding in *Cloudina*^3^ would rather support a cnidarian affinity for the cloudinids^6,28^. Bulbous and conical basal terminations have been documented from phosphatized specimens from South China^3,59^ and interpreted as evidence of direct development of a tubular structure from a rounded embryo and propagation via released daughter tubes with pointed terminations^59^. However, these developmental sequences do not exist in modern Cnidaria, where there is always a motile planula larva that hatches from an embryo, and tubular exoskeletons are only developed after larval settlement. When tubular exoskeletons are developed, they are attached to the substrate by a circular or more irregular basal disc (Supplementary Fig. 2C), which is distinct from the closed base sometimes documented in *Cloudina*. Indeed, tubes of modern annelids are mostly open at both ends in early development, but posterior tabulae can be developed, especially if the tube was damaged or the tube-dweller is disturbed^60^. It has been documented that annelids are not only capable of building tubular housings at the anterior end but also posteriorly, partly resulting in branched posterior tubes^51^. Although branching of modern serpulids does not exactly match the branching structures in some specimens of *Cloudina*^3,6^, it does indicate that annelids are capable of reproducing by clonal propagation and forming branched tubes^61^. Thus, the posteriorly closed tube, and the occurrence of branched tubes are not conclusive for assigning the biological affinity of cloudinids. However, further investigations are necessary to clarify the relationship and disparate taphomodes between the specimens of *Cloudina hartmannae* from South China, Spain, and Namibia.

Collared morphologies and smooth lumens are not reported in tube-forming cnidarians, which often have internal periderm teeth and basal circular attachment discs^57,62^. Instead, the tubular morphology of the cloudinids occurs more similarly in various families of tube-dwelling annelids. Furthermore, the fine lamellar ultrastructure with pseudo-orthogonal fabrics has been developed in several families of crown-group annelids. Delamination, as observed here in *Zuunia* gen. nov., is also typical during the decomposition of modern organic lamellate worm-tubes, such as siboglinids or alvinellids^31,41^. Although the Ediacaran tubular fossils represent only extracorporeal secretions and not the remains of organisms themselves, we conclude from morphological and ultrastructural evidences that the most parsimonious interpretation is to assign *Zuunia* nov. gen., and generally all cloudinids, with stem-group annelids. Our biological interpretation of Ediacaran cloudinids as tube-forming annelids is in agreement with previous estimates of the annelid-molluscan split between 590–540 my, based on molecular clock studies^63^.

## Methods

The phosphatic fossils from Mongolia were extracted with 10% buffered acetic acid following the procedure described in Yang *et al*.^64^. Organic lamellae and fibrous relic structures were released from the phosphatized tubes by applying 3% hydrochloric acid for about 20 minutes. Energy dispersive X-ray spectroscopy (EDS) and scanning electron microscopy (SEM) were conducted at the micro-laboratory of Continental Tectonics and Dynamics, Institute of Geology, Chinese Academy of Geological Sciences and Freie Universität Berlin. Samples were uncoated for the EDS analysis at 20 keV and high-resolution scanning. SEM images were taken at 20 keV with the samples coated by carbon or gold. Raman spectra were collected in the Micro-Raman Lab of IGCAGS with Horiba spectrometer LabRAM HR evolution equipped with an Olympus BX41 light microscope and calibrated by silicon wafer with 520.7 cm^−1^ Raman shift. Raman spectra were excited by 532 nm Nd: YAG laser with 100 mW laser power and received by 600 g optical grating through an 80 µm confocal hole. Nanofocus x-ray computed tomography was carried out at Technische Universität Dresden using a Phoenix Nanotom 180 kV. Cathodoluminescence (CL) analyses were carried out using a Reliotron III equipped with a Nikon Microscope at the College of Earth Science and Engineering, Shandong University of Science and Technology. The cathode voltage was set at 10–15 volts with an electric current at ~1 mA. CL images were taken under different exposure duration depending on the degree of luminescence. For comparative purposes, modern siboglinids collected from the Manus Basin (Site 33, depth 1741m, Voyage M054; N3°43′42.065″, E 51°40′20.851″) and the Okinawa Trough (depth 1361 m, Voyage R5; N 27°33.06116′, E 126°58.13521′). Samples of modern scyphozoan periderm tubes and hydrozoans for comparison of exoskeletal ultrastructure were freeze-dried with a CHRIST Alpha1-2LD freeze dryer at FUB and mechanically fractured.

## Supplementary information

Supplementary Information
