# Supplementary material for: Ultrastructure of Ediacaran cloudinids suggests diverse taphonomic histories and affinities with non-biomineralized annelids
Source: Sci Rep. 2020 Jan 17;10:535. doi: 10.1038/s41598-019-56317-x (PMC6968996; doi:10.1038/s41598-019-56317-x)
Supplement: Supplementary file 1 — Supplementary Information [file 41598_2019_56317_MOESM1_ESM.pdf]

Supplementary Information for

Ultrastructure of Ediacaran cloudinids suggests diverse taphonomic histories and affinities with non-biomineralized annelids

Ben Yang[1*], Michael Steiner[2*], James D. Schiffbauer[3, 4], Tara Selly[4, 3], Xuwen Wu[5], Cong Zhang[1, 6], Pengju Liu[1]

[1] MNR Key Laboratory of Stratigraphy and Palaeontology, Institute of Geology, Chinese Academy of Geological Sciences, Beijing 100037, China

[2] Department of Earth Sciences, Freie Universität Berlin, Berlin 12249, Germany

[3] Department of Geological Sciences, University of Missouri, Columbia, Missouri 65211, USA

[4] X-ray Microanalysis Core Facility, University of Missouri, Columbia, Missouri 65211, USA

[5] Laboratory of Marine Organism Taxonomy and Phylogeny, Institute of Oceanology, Chinese Academy of Sciences, Qingdao 266071, China

[6] School of Earth Science and Engineering, Shandong University of Science and Technology, Qingdao 266590, China

[*] E-mails: benyang@cags.ac.cn, michael.steiner@FU-Berlin.de

This PDF file includes:

Supplementary Figure 1 Simplified locality map of western Mongolia and generalized stratigraphic column with fossil distribution.

Supplementary Figure 2 Ultrastructure in exoskeletons of modern scyphozoans and hydrozoans.

Supplementary Figure 3 Energy dispersive X-ray analysis (EDX) of *Zuunia* gen. nov.

Supplementary Figure 4 Scatter plot of collar width vs. collar spacing in *Zuunia*, *Rajatubulus*, and *Cloudina*.

Supplementary Figure 5 Scatter plot of the collar widths and spacing of modern vestimentiferan tube worms.

Supplementary References

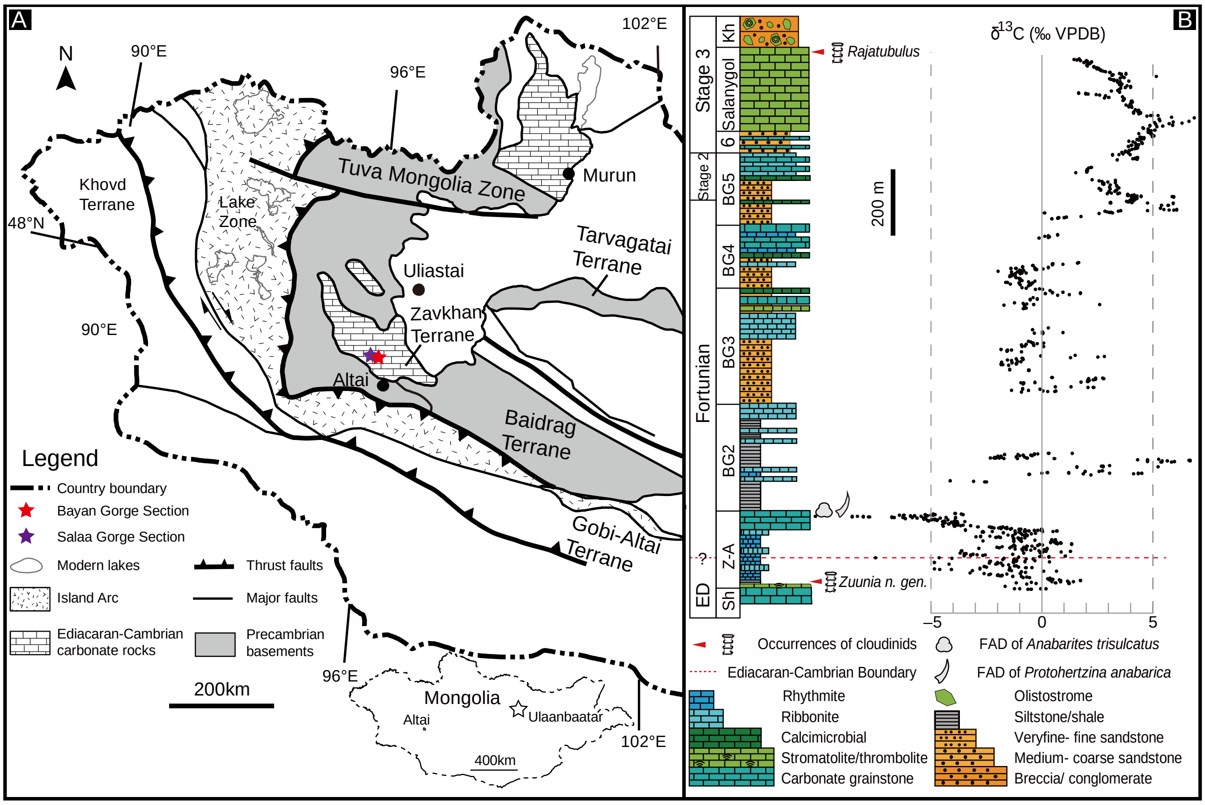

Supplementary Figure 1 (A), Simplified locality map of western Mongolia modified from Bold et al.[1] and Kröner et al. [2]. The red and violet stars respectively mark the Bayan Gorge (N46°41′52.9″, E96°18′22.7″) and Salaa Gorge (N46°48′32.1″, E95°46′18.8″) sections, Gobi-Altai Province, Mongolia. (B), Generalized stratigraphic column with the distribution of the studied fossils and the first appearance of small shelly fossils. Stratigraphic column and carbon isotope data are modified from Smith et al.[3]. Placement of the Ediacaran–Cambrian boundary is tentative according to Smith et al.[3]. BG, Bayangol Formation; ED, Ediacaran; Z-A, Zuun-Arts Formation; Sh, Shuurgat Formation; Kh, Khairkhan Formation.

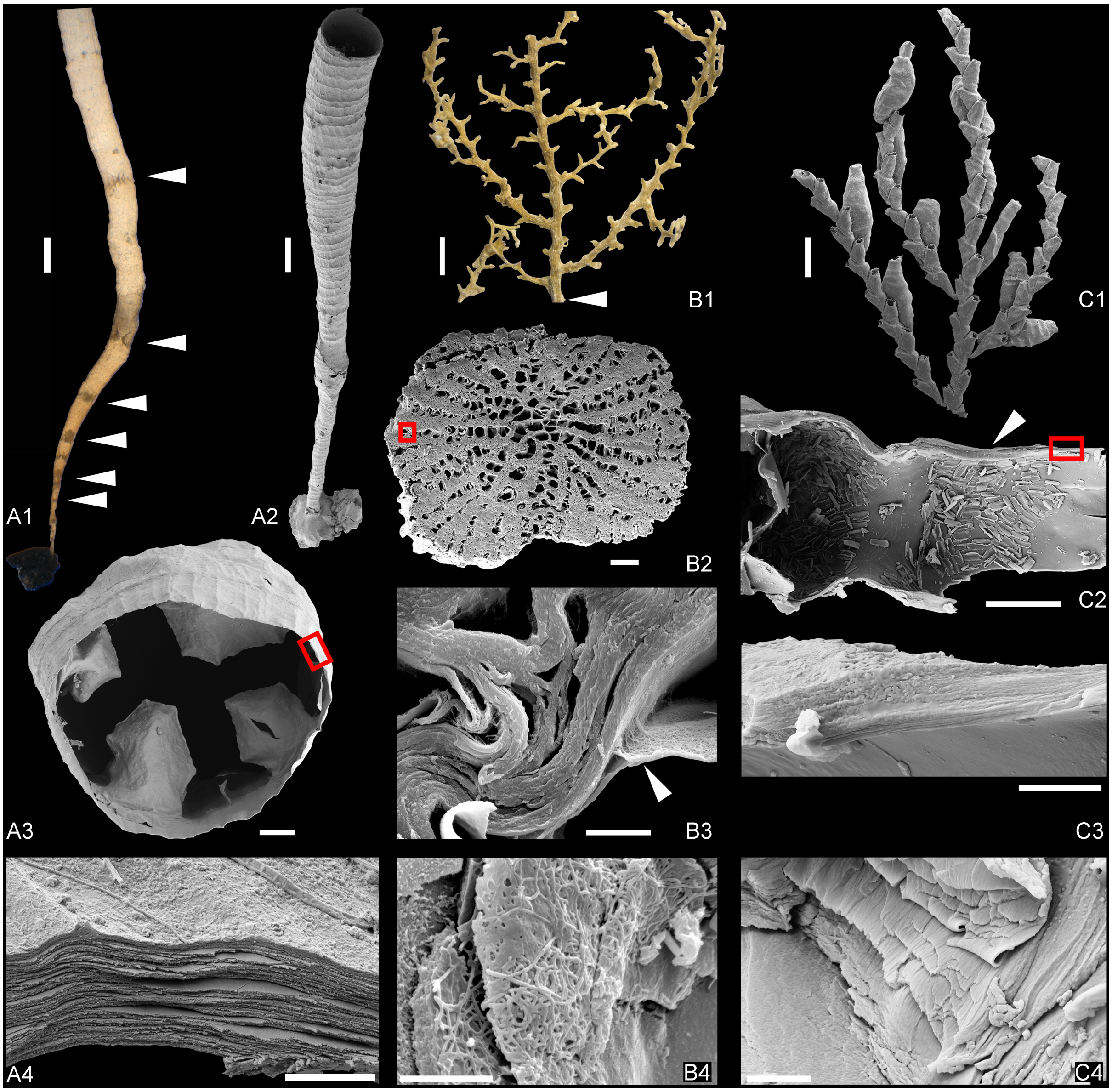

Supplementary Figure 2 Ultrastructure in exoskeletons of modern scyphozoans and hydrozoans. (A1), Photograph of modern Scyphozoan *Nausithoe werneri* under transmitted light (from cultivature at Hamburg University) showing rings of internal periderm cusps (white arrows). (A2) SEM photograph of (A1) showing the tube ornamentation and the basal attachment disc. (A3), SEM photograph of internal periderm teeth of A1. (A4), Close-up view of A3 (red frame) showing the massive lamellar structure of the wall of *Nausithoe werneri*. (B1), overview photograph of modern Hydrozoa *Stegolaria* sp. under reflected light. *Stegolaria* sp. from the seamount of Jiangpu Region, western Pacific, YS234-2. (B2), transverse view, SEM photograph of the hydrocaulus from B1 (red frame). (B3)， close-up of B2 (red frame) shows blocky structure of the hydrocaulus. (B4), close-up of B3 (white arrow), shows irregular

distribution of chitinous fibres on the inner surfaces of the cavities of hydrocaulus (B1). (C1), overview of SEM photography of *Seritularella* sp. from Alcoa Bay, South Africa, H1-Serit01a. (C2) a polyp opened by a lancet showing the layers of the theca and an inner side containing diatoms. (C3), close-up of the wall of C2 (red frame) showing that the wall is composed of massive organic lamellae. (C4), close-up of the wall of C2 (white arrow) showing lamellate structures. Scale bars: (B4, C4), 5 µm; (A4, B3, C3), 10 µm; (A3, C2), 100 µm; (A2, B2), 200 µm; (A1, C1), 1 mm; (B1), 2 mm.

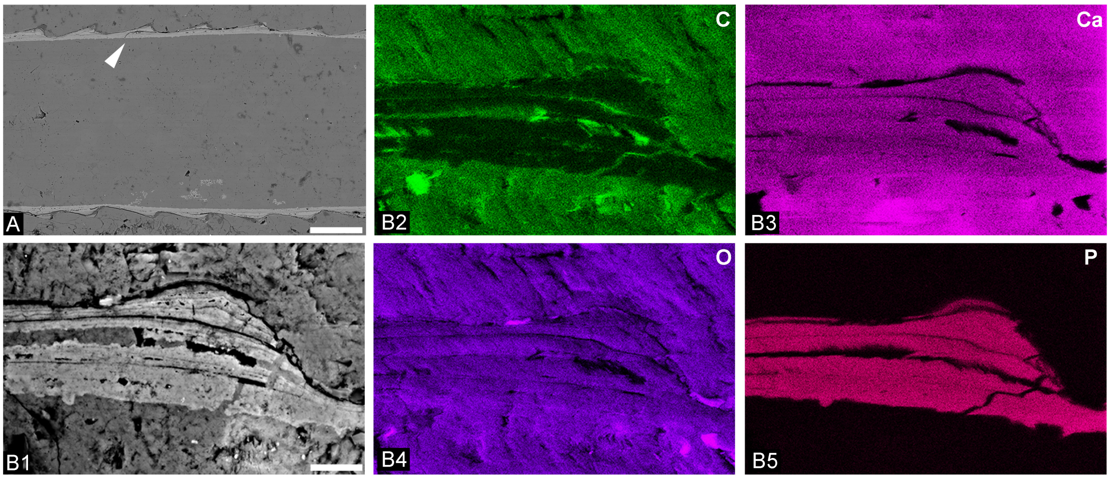

Supplementary Figure 3 Energy dispersive X-ray analysis (EDX) of *Zuunia* gen. nov. (A), back-scattered electrons (BSE) image of *Zuunia* gen. nov. in a thin section, coated with Pt, BYN1101. (B1), BSE image of one collar in (A, white arrow) without coating; note the fine, dark lamellae indicating carbon layers. (B2-5) EDX elemental maps of area shown in (B1), without coating. (B2), Carbon distribution. Note multiple fine carbon-rich layers in the collar. (B3), Calcium distribution. (B4), Oxygen distribution. (B5), Phosphorous distribution. Scale bars: (A), 100 µm; others, (B1-5), 10 µm.

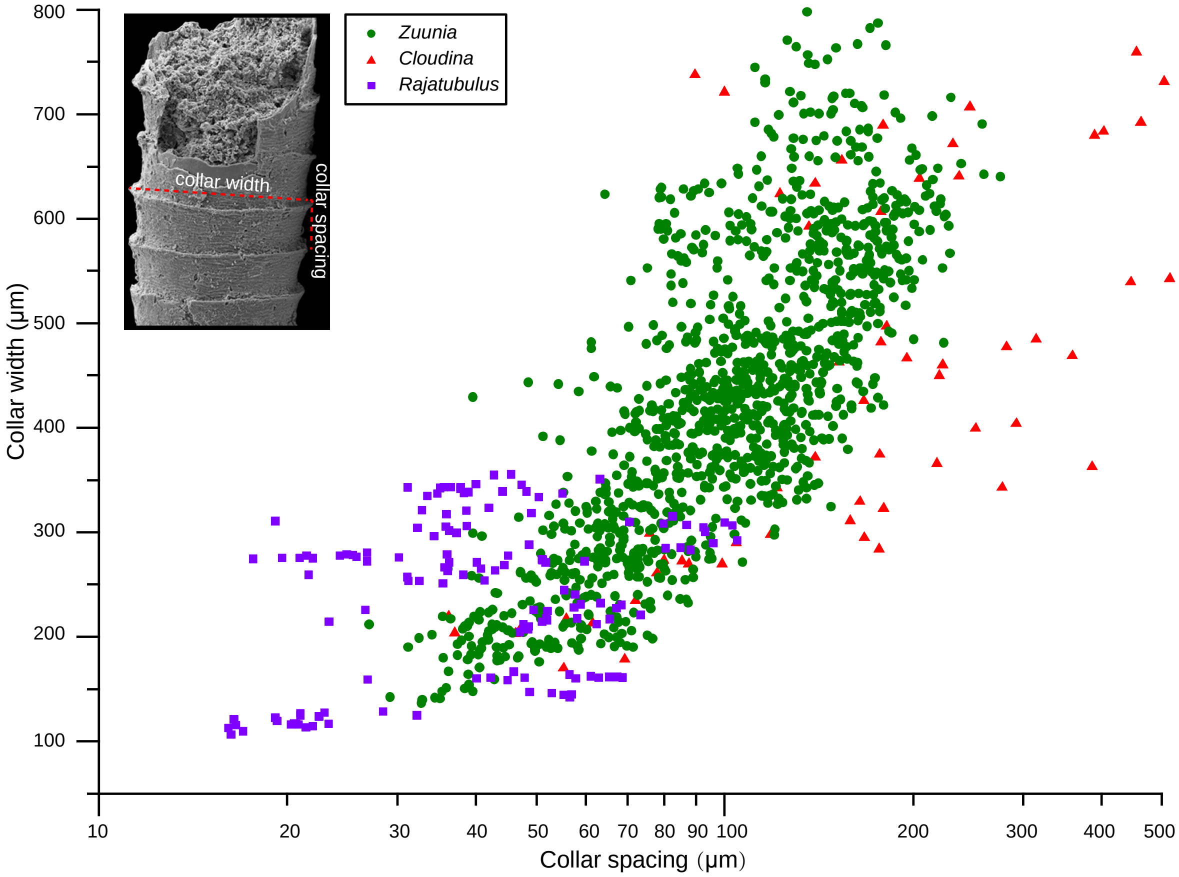

Supplementary Figure 4 Scatter plot of collar width vs. collar spacing in *Zuunia*, *Rajatubulus*, and *Cloudina*. Note the collar spacing is plotted logarithmically ($\log_{10}$) to highlight the distribution of the model. The data are restricted to a maximum collar distance of 500 μm. It is noted that the collar widths and spacing are relatively proportional for *Zuunia* and *Cloudina*. *Rajatubulus* shows two clusters of measurements, which may indicate the taxon potentially consists of more than one species.

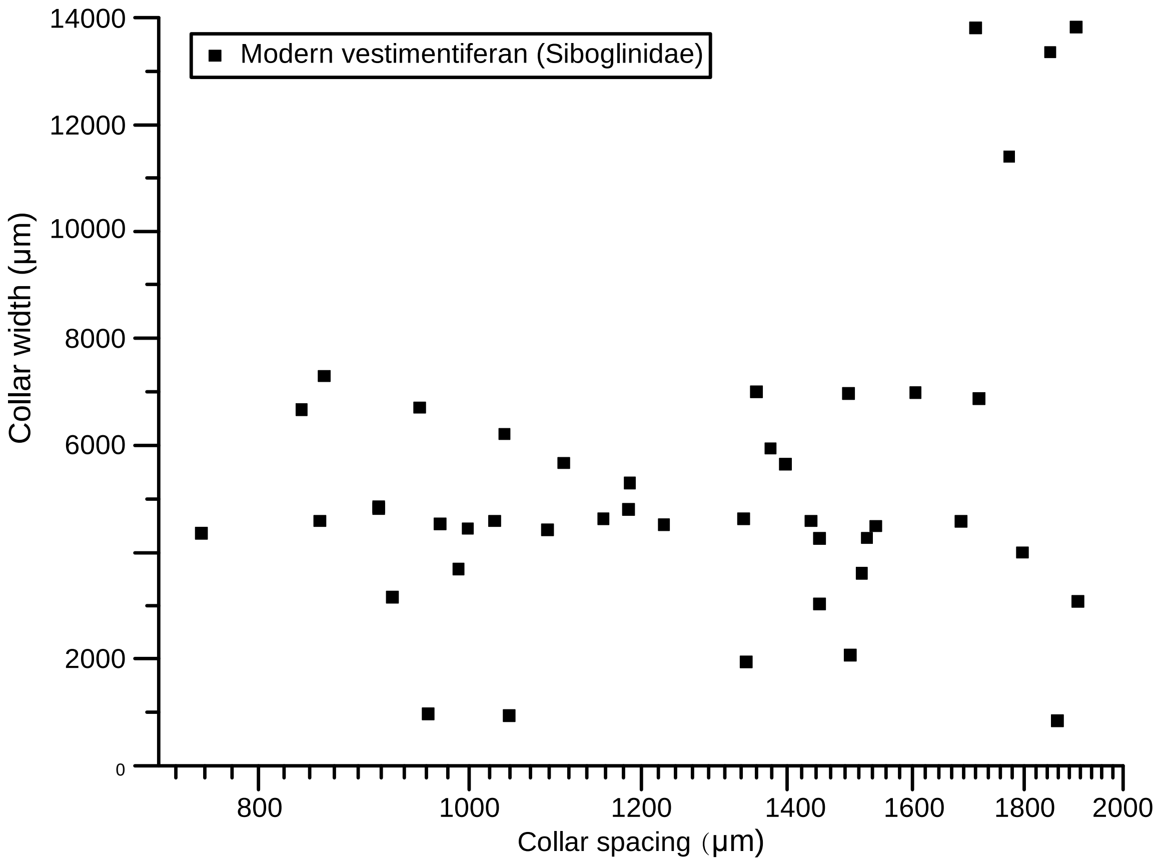

Supplementary Figure 5 Scatter plot of the collar widths and spacing of modern vestimentiferan tube worms. The data is restricted to a maximum collar distance of 2000 μm. The plot shows little correlation between the collar widths and spacing, which is in accordance with the observation of the specimen of *Tevnia jerichonana* from Smithsonian (USNM Number NC400407-DSP) .

Supplementary References

1. Bold U, Crowley JL, Smith EF, Sambuu O, Macdonald FA. Neoproterozoic to early Paleozoic tectonic evolution of the Zavkhan terrane of Mongolia: Implications for continental growth in the Central Asian orogenic belt. *Lithosphere* **8**, 729-750 (2016).

2. Kröner A, *et al.* Zircon ages for a felsic volcanic rock and arc-related early Palaeozoic sediments on the margin of the Baydrag microcontinent, central Asian orogenic belt, Mongolia. *Journal of Asian Earth Sciences* **42**, 1008-1017 (2011).

3. Smith EF, Macdonald FA, Petach TA, Bold U, Schrag DP. Integrated stratigraphic, geochemical, and paleontological late Ediacaran to early Cambrian records from southwestern Mongolia. *Geol Soc Am Bull* **128**, 442-468 (2016).
